# Development of a social learning theory-based pressure injury training program for nursing assistants in Chinese nursing homes

**DOI:** 10.3389/fpubh.2024.1478147

**Published:** 2025-02-07

**Authors:** Yanxia Guo, Shengnan Yang, Plernpit Boonyamalik, Arpaporn Powwattana, Wen Zhu, Lingxia Xu

**Affiliations:** ^1^School of Nursing and Midwifery, Jiangsu College of Nursing, Huaian, China; ^2^Department of Public Health Nursing, Faculty of Public Health, Mahidol University, Bangkok, Thailand; ^3^Faculty of Nursing, Jiangsu Health Vocational College, Nanjing, China; ^4^Department of Neurology, Yancheng Third People’s Hospital, Yan Cheng, China

**Keywords:** pressure injury, training program, nursing assistants, Delphi method, social learning theory, nursing homes

## Abstract

**Introduction:**

Pressure injury (PI) is a significant concern in Chinese nursing homes, particularly in China, especially due to the rapidly aging population. Nursing assistants play a vital role in PI prevention and management but often lack adequate training. To address this gap, we developed a training program for nursing assistants based on Social Learning Theory (SLT), aimed at improving their competencies in PI prevention and management. The modified Delphi method was used to gather expert consensus on the program’s structure and content.

**Methods:**

A two-round Delphi process was performed involving an expert panel in wound care, community nursing, geriatric nursing, and nursing education. The training program was designed based on SLT, emphasizing observational learning, enactive learning, and behavioral reinforcement. Several experts evaluated the training program’s content, which was informed by systematic reviews and qualitative interviews with stakeholders. Data analysis included expert’s positive coefficient, expert’s authority coefficient (Cr), expert’s coordination coefficient (Kendall’s W), and coefficient of variation (CV) were used to reflect reliability and consensus.

**Results:**

Consensus was reached on 79 key indicators for the training program, which included 4 first-level indicators (training objectives, content, methods, and evaluation), 13 second-level indicators, and 62 third-level indicators. The expert authority coefficient was 0.93, and Kendall’s W values of 0.372 (*p* < 0.001) in the first round and 0.177 (*p* < 0.001) in the second round indicated strong agreement among experts. The program integrates SLT principles, such as attention, retention, motor reproduction, and motivation, to enhance the training’s effectiveness.

**Conclusion:**

The study developed a comprehensive SLT-based PI training program for nursing assistants in Chinese nursing homes using the modified Delphi method. The program addresses the critical need for competency-based training in PI prevention and management. Future research should focus on the implementation and evaluation of this program in real-world settings to determine its effectiveness in improving nursing assistants’ skills and reducing PI incidence among older adult residents.

## 1 Introduction

Pressure injury/injuries (PI/PIs), also referred to as bedsores, decubitus, pressure ulcers, and pressure sores. It is described as localized damage caused by pressure or pressure combined with shear to the skin and/or underlying tissue (1). PIs are one of the most frequently occurring and costly, yet preventable adverse events in institutions and are of particular concern for older adults (2), which are a well-recognized complication of nursing homes (3). In China, the aging population is rapidly increasing, leading to a growing demand for long-term care facilities. By 2020, more than 254 million Chinese people were 60 years of age or older, making up 18.1% of the total population (4). This demographic shift underscores the urgent need for effective pressure injury prevention and management (PIPM) strategies in nursing homes (5). Studies showed that the prevalence of PIs in nursing homes varied from 3.4 to 32.4% globally (6), and the incidence of PIs in nursing homes varied from various countries being reported to range from 3.6 to 39.4% (7). It was reported 1.91–10.4% and 28.9% for PI prevalence and incidence in Chinese nursing homes (8, 9). PIs could trigger a tremendous burden not only on individuals but also on the healthcare systems (10).

Nursing assistants are the primary workforce and caregivers in nursing homes and are essential members of the frontline team who devote a lot of time observing and tracking the outcomes of long-term care residents (11). Therefore, nursing assistants play a crucial role in PIPM. Nursing homes are placing demands on qualified nursing assistants with positive attitudes, enhanced knowledge, and the requisite skills to deliver high-quality care services, ultimately improving the quality of life for residents (12). Bangova pointed out that insufficient knowledge or improper care behavior of nursing assistants will increase the incidence of PIs to a certain extent (13). Due to their likely insufficient knowledge and skills, a large number of nursing assistants initiated preventive care after PI was discovered, inappropriately used PI prevention materials, or performed inappropriate PIPM practices (14). Although guidelines have been provided over the years, of which this information is key when assessing skin breakdown or precursors to skin breakdown, studies showed it is rarely taught to the nursing assistants who are the personnel most likely to assess the beginning signs of skin breakdown (14). Lavallée et al. (15) conducted interviews with nursing home staff, and they identified knowledge as one barrier to PI prevention (15).

To ensure nursing assistants are competent in their work, they need to undergo an innovative education or training program to bridge the knowledge-to-action gap in nursing practice (16). International evidence has consistently identified that training can improve nursing assistants’ competencies in PIPM, strengthen the cognition toward diseases and attitude toward work, as well as promote better care and outcomes for the older adult in nursing homes (17). The Chinese Health Commission launched an advocacy on strengthening the training and standardized management of nursing assistants in 2019, which mentioned that institutions should use trained and qualified nursing assistants to engage in corresponding work, and require active training based on the training outline (18). However, currently, the limited PI-specific training programs mainly target nurses, patients, or family caregivers, and the training curriculum quality varies greatly between these programs worldwide (19, 20). There is a lack of high-quality PI training programs focused on nursing assistants working in nursing homes, especially targeted at enhancing nursing assistants’ competencies around the world. In the UK, there is no national education program for nursing assistants designed to improve skills related to skin assessment and PI prevention (21). In the USA, Wogamon (14) developed a PI training program for nursing assistants in a nursing home, but the program just included some simple content, such as PI causes, risk factors, stages, positioning, documentation, and reporting of pertinent data which were based on the outdated guideline NPUAP/EPUAP 2011. Besides, the training hours were only 2 h with lectures. In Sweden, Hultin et al. (22) trained the nursing assistants from the older adult care facility to improve their PIPM competencies mainly targeting the use of a pressure mapping system based on the outdated guideline NPUAP/EPUAP 2014. The training methods included 15 min training and 1 week of self-practice. All of these programs were not designed based on a theoretical framework. Moreover, in a review of 24 previous studies of comprehensive PI prevention programs (23), 20 of which were of programs in acute care settings and four in long-term care facilities, and most participants were nursing staff not just focused on nursing assistants. Therefore, it is very imperative and urgent to develop a comprehensive, evidence-based, theory-based, innovative training methods-based effective PI training program for nursing assistants.

Social Learning Theory (SLT) was developed by Albert Bandura (1977), and it was widely applied to various training programs in the public health field as the theoretical framework to enhance healthcare providers’ competencies (24). Applying SLT in medical education can strengthen learning behavior and solve common clinical teaching problems through learners’ observation and demonstration (25). Traditional educational models often fail to provide the necessary interactive and observational learning experiences that are essential for nursing assistants to retain and apply critical knowledge in PIPM (26). The SLT-informed model offers a practical framework to bridge this gap. The SLT is a theory of learning that takes into account how individuals learn from each other as well as how learning takes place in social contexts (27). Central to SLT are self-efficacy, knowledge, and skills. By integrating SLT into nursing training, educators can create a dynamic and effective learning environment that prepares future healthcare providers to excel in their profession (28). According to the SLT, the learning process of nursing assistants can take place through observation, imitation, and modeling from experts, which are regulated by four processes namely attention (e.g., paying attention to the online and offline learning), retention (e.g., knowledge quizzes, rehearsal practice, etc.), motor reproduction (e.g., practice feedback), and motivation (e.g., giving positive feedback, reduced workload, improved income, improved working conditions, etc.) process (29). SLT is consistent with the traditional behaviorists’ opinions that they insist upon the importance of practice and repetition in learning and positive or negative reinforcement can be used to encourage the repetition of the behavior (30). The modified Delphi method, an organized and iterative procedure that experts use to come to an agreement, provides a robust framework for developing a training program tailored to the needs of nursing assistants. This method involves multiple rounds of surveys or questionnaires, allowing experts to refine their opinions and achieve consensus on specific topics (31).

This study aims to develop a comprehensive PI training program based on SLT for nursing assistants in Chinese nursing homes using the modified Delphi method. This study can contribute to the broader goal of enhancing geriatric care and promoting the health and wellbeing of the aging population in China. In addition, This study is not only valuable for improving the quality of nursing care in Chinese nursing homes, but also has far-reaching implications for nursing practice and health policy making worldwide.

## 2 Materials and methods

### 2.1 Study design

**Figure 1:**
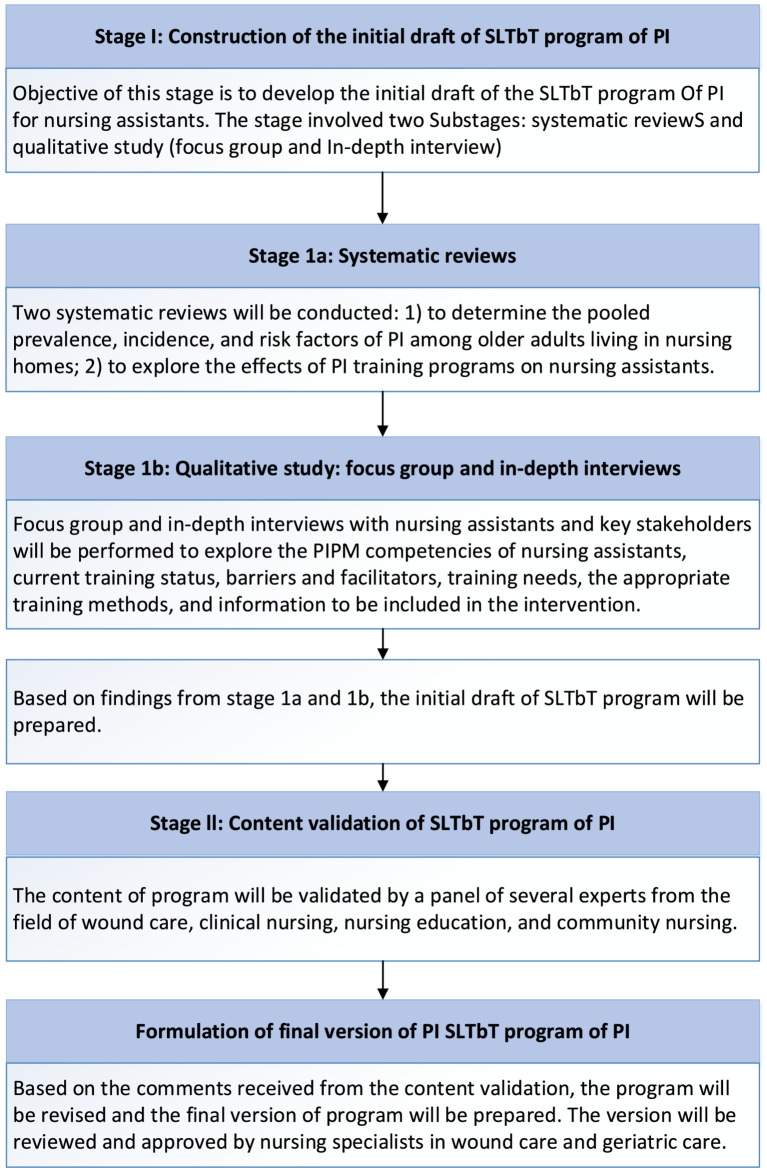
Process of program development.

A modified two-round Delphi technique was employed to reach a consensus among a panel of experts with extensive PIPM experience. The basic idea behind the Delphi method is to use the qualitative evaluation of the evidence to derive quantitative estimates (32, 33). The development process of the SLT-based training (SLTbT) program of PI was divided into two stages: the stage of the initial draft construction of the SLTbT program by systematic reviews and qualitative study; the stage of content validity of expert panel review (Delphi Method) to form the final version of the SLTbT program. All stages were conducted between July and August 2024. The specific development process is shown in Figure 1. After identifying the research issue, the research team compiled the initial version draft of the SLTbT program of PI informed by the information acquired from the systematic reviews, focus group, and in-depth interviews. The content validation of the initial program was validated by the expert panel review approach (Delphi technique) to finalize the components of the SLTbT program by achieving a consensus from several designated experts. Based on the experts’ opinions or comments received from the content validation, the training program was revised as a final version of SLTbT program. Delphi method does not need researcher to convene the panel experts to meet face-to-face rather communication with the researcher through email, to ensure anonymity and privacy and allow each freedom of expression without outside pressure or influence (33, 34). It involved in using questionnaires to obtain the collective opinion of experts until a consensus is reached (35).

### 2.2 Delphi method process

The research team confirmed the initial draft of the SLTbT program including 78 indicator items based on the systematic review, and qualitative study, and entered into the modified Delphi process.

#### 2.2.1 Panel member recruitment and data collection

An expert panel consisting of several content experts was invited to evaluate the appropriateness, and feasibility of the SLTbT program components using the Delphi technique with two rounds. Between July and August 2024, 15 experts were invited to participate in this study via email or WeChat. Each questionnaire was required to be completed by experts within 2 weeks and returned to the researcher. The experts were selected through purposeful sampling. The inclusion criteria for participants were: (1) come from the hospitals, community healthcare centers, or nursing educational institutions, with much expertise in PIPM; (2) have at least 5 years of wound care experience or at least 5 years of nursing teaching experience; (3) have an associate senior title or higher; (4) have rich research experience in PI field and geriatric care; (5) have a bachelor’s degree or higher. Experts who satisfied the inclusion requirements were invited to join the expert panel by sending emails or using WeChat (34). The participants who are not willing to participate in this study or those who are not interested in the study are excluded. The size of the Delphi expert panel is usually 10–15 members (36).

#### 2.2.2 Consultation questionnaire

The SLTbT program was presented in the form of a consultation questionnaire, including three parts: introduction, expert characteristics, and appropriateness assessment of the PI training program. The initial draft of SLTbT program included 78 indicator items: 4 first-level indicators involving 4 domains (training objectives, training contents, training methods, training evaluation), 13 s-level indicators (three indicators for training objectives domain, two indicators for training contents domain, three indicators for training methods domain, five indicators for training evaluation domain), and 61 third-level indicators (27 indicators for training objectives domain, 21 indicators for training contents domain, eight indicators for training methods domain, five indicators for training evaluation domain). The appropriateness assessment used a 5-point Likert scale to rate responses for each indicator, where 1 means “not at all appropriate” and 5 means “very appropriate.” Furthermore, a column of modification opinions has been attached for experts to add, delete, and revise. The mean value and the coefficient of variation (CV) of appropriateness assignment can be evaluated to assess the experts’ agreement degree of each indicator (37).

#### 2.2.3 Delphi round 1

The researcher sent the initial consultation questionnaires to all experts’ emails or WeChat and received their response within 2 weeks. Then the researcher summarized and analyzed the experts’ opinions. Every item that satisfied the consensus filtering criteria was kept in the program. All the consensus items and the revised items based on experts’ opinions were entered into Delphi round 2.

#### 2.2.4 Delphi round 2

Experts rated their degree of agreement with each item statement once more in the Delphi round 2. The consultation was terminated when the expert panel came to a consensus following two rounds of the Delphi study.

### 2.3 Data analysis

We used Microsoft Excel 2010 and IBM SPSS 20.0 software to extract and analyze data. Descriptive analysis was used for the basic information of experts. The scientific soundness and rationality of the Delphi method are reflected by three indicators: experts’ positive coefficient, authority coefficient, and coordination coefficient (33).

1. The experts’ positive coefficient indicates the level of experts’ interest in the study and is represented by the response rate of the expert consultation (38). It demonstrated the experts’ strong enthusiasm for the research when it was >70%.
2. Experts’ authority coefficient (Cr) represents the reliability of the consultation results (37). Expert judgment (Ca) and expert familiarity with the research topic (Cs) were used to calculate the Cr. The formula is (Ca + Cs)/2, and a value of Cr ≥0.70 is deemed acceptable reliability (39, 40). Theoretical analysis, practical experience, knowledge from domestic and foreign literatures, and intuition served as the foundation for Ca, and the high, middle, and low levels were used to reflect the influence degree of judgment basis. The details are as follows: theoretical analysis (0.3, 0.2, 0.1), practical experience (0.5, 0.4, 0.3), knowledge from domestic and foreign counterparts (0.1, 0.1, 0.05), and intuition (0.1, 0.1, 0.05) (See Table 1). The levels of Cs were categorized ranging from “very familiar” to “very unfamiliar,” with corresponding values of 1.0, 0.8, 0.6, 0.4, and 0.2, respectively (40, 41).
3. The coefficient of variation (CV) is used to reflect the degree of consensus among experts’ ratings of each indicator. The smaller the CV value, the better the consensus among experts’ evaluations (37). The value is 0–1. The calculation formula of CV for each indicator item is SD/mean, and the mean value >4.0 or CV <0.25 is the consensus criteria for filtering items (37).

**Table 1:** Judgment basis with the topics for consultation from experts.

Evaluation criteria of Ca	Degree of judgment basis		
	High	Middle	Low
Theoretical analysis	0.3	0.2	0.1
Practical experience	0.5	0.4	0.3
Knowledge from domestic and foreign literatures	0.1	0.1	0.05
Intuition	0.1	0.1	0.05

### 2.4 Ethical consideration

This study was reviewed by the institutional review board at Mahidol University in Thailand (IRB number: MUPH 2024–066) and Jiangsu College of Nursing in China (IRB number: JSCN-ME-2024071801). All participants voluntarily participated in this study.

## 3 Results

### 3.1 Expert information

The study recruited 15 experts in each Delphi round including 13 women and 2 men. The age range was 35–52 (39.87 ± 5.514) years old. These experts came from tertiary hospitals (*n* = 8, 6 wound care specialists, and 2 geriatric care specialists), community healthcare centers (*n* = 4), and nursing educational institutions (*n* = 3). The working experience range was 9–30 (15.47 ± 7.53) years. Five experts held bachelor’s degrees, eight held master’s degrees, and two held doctoral degrees. Eleven experts had associate senior titles, and four had senior titles. All the experts were experienced in the PI field; of them, four worked in community nursing, three in nursing education, and eight in clinical nursing (6 as wound care specialists and 2 as geriatric care specialists).

### 3.2 Experts’ positive coefficient

Consultation questionnaires were issued to 15 experts in each round, and the effective response rate was 100.00%. The positive coefficient was higher than 70%, indicating that the experts had a high level of enthusiasm in this study.

### 3.3 Experts’ authority coefficient

In this study, Expert judgment (Ca) = 0.98, expert familiarity (Cs) = 0.87, and Cr = 0.93, so the results are highly reliable.

### 3.4 Experts’ coordination coefficient

Kendall’s W represents the experts’ coordination coefficient. In the first round, the Kendall’s W was 0.372 (*p* < 0.001, *χ*^2^ = 429.489). In the second round was 0.177 (*p* < 0.001, *χ*^2^ = 207.472). The results showed that there is a high degree of agreement between the experts’ opinions.

### 3.5 Selection of indicators in the first round

The mean, standard deviation, and coefficient of variation (CV) of each indicator’s expert ratings were calculated based on the results of the first Delphi round. In this round, the CV range for each indicator was 0.05–0.53, and the mean range was 2.00–4.93. Three indicators (A117, A123, A124) had CV > 0.25 and means <4.0, so these three indicators were deleted. Detailed statistical results of the indicators are shown in Table 2. In the first round, 6 experts proposed modifications to 14 indicators. Nine indicators were modified, three indicators were added and two indicators were merged. The specific modifications are shown in Table 3.

**Table 2:** Statistical results of expert consultation for the first round indicators.

Indicator items	Mean	Standard deviation	CV
A1 Training objectives	4.93	0.258	0.052338
A11 Knowledge objectives	4.93	0.258	0.052338
A111 Master PI definition	4.87	0.352	0.072301
A112 Understand PI epidemiological characteristics and hazards	4.60	0.507	0.110238
A113 Understanding PI mechanisms	4.20	0.775	0.184428
A114 Understand PI staging and clinical manifestations	4.53	0.640	0.141163
A115 Understand PI risk factors	4.93	0.258	0.052338
A116 Understand the population and sites prone to PI	4.93	0.258	0.052338
A117 Understand PI characteristics in the older adult	3.20*	1.320	0.412554*
A118 Familiarize with PI characteristics and causes in nursing homes	4.73	0.458	0.096705
A119 Familiarize with PI common types and selection of dressings associated	4.20	0.862	0.205212
A1110 Learn how to differentiate PI from other wounds such as incontinence-associated dermatitis and diabetic foot ulcers	4.00	0.845	0.211289
A1111 Understand the types and functions of common pressure relief equipment	4.07	0.704	0.173049
A1112 Familiarize with the key points of nutrition care	4.87	0.516	0.106109
A1113 Master skin care methods	4.87	0.352	0.072301
A1114 Understand PI common treatments	4.40	0.632	0.14374
A12 Skill objectives	4.87	0.352	0.072301
A121 Be able to correctly determine PI clinical stage, especially correctly identify stage I PI	4.67	0.617	0.13226
A122 Learn to use the Braden PI Risk Assessment Tool	4.07	0.884	0.217307
A123 Learn to master effective PI prevention and management techniques by observing and imitating the behaviors of teachers and excellent caregivers	2.93*	0.961	0.327665*
A124 Develop the ability to assess PI risk and develop a personalized prevention plan	2.00*	1.069	0.534522*
A125 Learn proper nursing skills, including moving and turning techniques, to reduce PI occurrence	4.93	0.258	0.052338
A126 Learn simple wound care techniques	4.07	0.884	0.217307
A13 Attitude objectives	4.93	0.258	0.052338
A131 Be able to respect and care for the older adult and love older adult care work	4.93	0.258	0.052338
A132 Be able to understand the laws and regulations related to PI in nursing homes, and respect and protect the legal rights and interests of the older adult	4.87	0.352	0.072301
A133 Through active participation in training courses, enhance self-efficacy, build the right attitude and confidence in preventing and managing PI	4.87	0.352	0.072301
A134 Cultivate teamwork spirit, share experience and skills with colleagues, and jointly improve the quality of care	4.93	0.258	0.052338
A135 Develop a high level of concern for the physical and mental health of the older adult, and always maintain patience, care and responsibility.	4.67	0.617	0.13226
A136 Be familiar with the characteristics of the older adult, master the skills of communicating with the older adult, and provide psychological care	4.80	0.414	0.086258
A137 Learn to communicate effectively with nurses and be able to work together to solve patient problems	4.73	0.594	0.125412
B1 training content	4.93	0.258	0.052338
B11 Theoretical knowledge module	4.73	0.594	0.125412
B111 Professional ethics and code of conduct for nursing home caregivers	4.93	0.258	0.052338
B112 Legal and ethical issues related to older adult care and PI	4.80	0.561	0.116794
B113 Definition and epidemiological characteristics of PI	4.40	0.507	0.115248
B114 The dangers of PI	4.93	0.258	0.052338
B115 Causes and risk factors for PI	4.87	0.352	0.072301
B116 Staging and clinical manifestations of PI	4.60	0.507	0.110238
B117 Causes and characteristics of PI among the older adult in nursing homes	4.93	0.258	0.052338
B118 Methods for risk assessment of PI	4.27	0.594	0.139129
B119 Differentiate PI from other common skin problems	4.20	0.676	0.160982
B1110 Nutrition care	4.73	0.458	0.096705
B1111 Skin care	4.93	0.258	0.052338
B1112 Treatment and management of PI	4.33	0.816	0.188422
B1113 Types of pressure relief equipment	4.07	0.884	0.217307
B1114 Dressing selection and use	4.00	0.756	0.188982
B1115 Psychological care for patients with PI	4.60	0.507	0.110238
B1116 Communication skills for PI	4.80	0.561	0.116794
B12 practical knowledge module	4.80	0.561	0.116794
B121 Repositioning skills	4.93	0.258	0.052338
B122 Position transfer techniques	4.93	0.258	0.052338
B123 Wound dressing method and process	4.20	0.775	0.184428
B124 PI risk assessment methods and process	3.27*	1.100	0.336668*
B125 Use of walking aids	4.47	0.743	0.166393
C1 training methods	4.87	0.352	0.072301
C11 training format	4.80	0.561	0.116794
C111 Online theoretical knowledge training	4.73	0.594	0.125412
C112 Offline practical skills training	4.93	0.258	0.052338
C12 Teaching methods	4.87	0.352	0.072301
C121 Attention: Online self-learning by watching videos + offline learning by observing and imitating role models (expert workshops + case teaching);	4.80	0.414	0.086258
C122 Retention: Online knowledge test + offline independent practice + offline scenario simulation + offline peer learning + offline cooperative learning;	4.93	0.258	0.052338
C123 Reproduction: Knowledge feedback (theory and skills feedback and real-time feedback)	4.60	0.507	0.110238
C124 Motivation: encouraging teaching (giving positive feedback), policy support (such as reduced workload, improved income, improved working conditions)	4.53	0.640	0.141163
C13 Training time	4.53	0.640	0.141163
C131 Online course training: Each video is <10 min long, with a total of 10–15 videos, and can be completed within 2 weeks	4.80	0.561	0.116794
C132 Offline training: 2 h of teaching demonstration and 3 h of independent practice, completed within 1 week.	4.60	0.632	0.13749
D1 training evaluation	4.93	0.258	0.052338
D11 knowledge evaluation	4.93	0.258	0.052338
D111 Use the “PI Knowledge Questionnaire” designed by Zhou Dongmei, measuring the results pre-training and post-training.	4.93	0.258	0.052338
D12 Skills evaluation	4.93	0.258	0.052338
D121 Use the “PI Practice Questionnaire” designed by Zhou Dongmei and “PI identification skills questionnaire (including PI staging and differentiation from other skin problems) (self-designed),” measuring the results pre-training and post-training.	4.87	0.352	0.072301
D13 Attitude evaluation	4.67	0.488	0.104561
D131 Zhou Dongmei version of the PI Attitude Questionnaire and the general self-efficacy scale will be used, measuring the results pre-training and post-training.	4.93	0.258	0.052338
D14 Process evaluation	4.53	0.516	0.113911
D141 During the training process, the online in-class tests and offline independent practice effects will be evaluated.	4.80	0.414	0.086258
D15 Satisfaction evaluation	4.93	0.258	0.052338
D151 After the training, the nursing assistants evaluated their satisfaction and gave feedback on the training format, teaching methods, time schedule, teachers, and content, etc.	4.93	0.258	0.052338

**Table 3:** Summary of modifications from expert consultation in the first round.

Indicator items	Experts’ opinions	Modifications
Firs-level indicator		
Added indicator	One expert suggested: “Training Time” should be considered as a primary indicator, rather than being placed under “Training Methods” as a secondary indicator.	After discussion within the research team, it was decided not to agree with the modification.
Second-level Indicator	No suggestions for modification	
Third-level Indicator		
1.1.11 Understand the types and functions of common pressure relief equipment	One expert suggested: Change “understand” to “be familiar with.”	Agree and make the change
1.2.5 Learn proper nursing skills, including moving and Repositioning techniques, to reduce PI occurrence	One expert suggested: Divide the objectives into smaller skill objectives, such as learning to transfer positions, learning to turn over, learning to use a walking aid, etc.	Agree and make the change
1.2.6 Learn simple wound care techniques	Two experts suggested: Modify it to “Understand the technique and process of wound dressing change.”	Agree and make the change
1.3.6 Be familiar with the characteristics of the older adult, master the skills of communicating with the older adult, and provide psychological care	One expert suggests: Merge the two items into one indicator, and modify it to “Master the skills of communication with nurses, the older adult, and their families, and provide psychological care for the older adult.	Agree and make the change
1.3.7 Learn to communicate effectively with nurses and be able to work together to solve patient problems		
2.1.5 Causes and risk factors for PI	One expert suggested: Modify it to “The mechanism and risk factors of PI”	Agree and make the change
2.1.9 Differentiate PI from other common skin problems	Two experts suggested: It is necessary to provide a detailed explanation on what are the other common skin problems.	Agree and make the change
2.1.3 Types of pressure relief equipment	Two experts suggested: Modify it to “Types and functions of pressure relief equipment.”	Agree and make the change
Added Indicator	Two experts suggested: Add a third-level indicator item under the secondary indicator “Theoretical Knowledge Module” for “High-risk groups and common body sites for PI”	Agree and make the change
Added Indicator	One expert suggests: Add a third-level indicator item under the secondary indicator “Skill Knowledge Module” for “The selection and usage methods of dressings.”	Agree and make the change
3.1.1 Online theoretical knowledge training	Two experts suggested: It is recommended to provide a detailed description of how to ensure that the video is completed in online learning.	Agree and make the change
3.3.2 Offline training: 2 h of teaching demonstration and 3 h of independent practice, completed within 1 week.	Two experts suggested: It is recommended to provide a detailed explanation of the self-practice schedule arrangement.	Agree and make the change
4.2.1 Zhou Dongmei version of the PI Care Practice Questionnaire and PI identification (including PI staging and differentiation from other skin problems) skills questionnaire (self-designed) will be used to compare the results before training and with the control group.	One expert suggested: Split the two questionnaires into two separate indicators.	Agree and make the change

### 3.6 Selection of indicators in the second round

The research team made revisions and formed a second round of expert consultation questionnaires based on the results of the first round. The experts’ opinions tended to be consistent after the second round. The appropriateness scores of each indicator reached the inclusion criteria, the CV range of each indicator was 0.05–0.20, and the mean for each indicator was >4. The detailed statistical results of the indicators are shown in Table 4. What is more, the experts did not add, delete, or modify any indicators, nor did they make any other specific suggestions. The SLTbT program of PI included 79 indicator items ultimately: 4 first-level indicators involving 4 domains (training objectives, training contents, training methods, training evaluation), 13 second-level indicators (three indicators for training objectives domain, two indicators for training contents domain, three indicators for training methods domain, five indicators for training evaluation domain), and 62 third-level indicators (25 indicators for training objectives domain, 22 indicators for training contents domain, eight indicators for training methods domain, seven indicators for training evaluation domain).

**Table 4:** Statistical results of expert consultation for the second round indicators.

Indicator items	Mean	Standard deviation	CV
A1 Training objectives	4.87	0.352	0.07
A11 Knowledge objectives	4.93	0.258	0.05
A111 Master PI definition	4.87	0.352	0.07
A112 Understand PI epidemiological characteristics and hazards	4.40	0.828	0.19
A113 Understanding PI mechanisms	4.20	0.775	0.18
A114 Understand PI staging and clinical manifestations	4.53	0.640	0.14
A115 Understand PI risk factors	4.60	0.910	0.20
A116 Understand the population and sites prone to PI	4.73	0.799	0.17
A117 Familiarize with the characteristics and causes of PI occurring in nursing homes.	4.13	0.834	0.20
A118 Familiarize with PI common types and selection of dressings associated	4.60	0.632	0.14
A119 Learn how to differentiate PI from other wounds such as incontinence-associated dermatitis and diabetic foot ulcers	4.20	0.862	0.21
A1110 Understand the types and functions of common pressure relief equipment	4.00	0.845	0.21
A1111 Familiarize with the key points of nutrition care	4.07	0.704	0.17
A1112 Master skin care methods	4.73	0.594	0.13
A1113 Understand PI common treatments	4.73	0.594	0.13
A12 Skill objectives	4.87	0.352	0.07
A121 Be able to correctly determine PI clinical stage, especially correctly identify stage I PI	4.53	0.743	0.16
A122 Learn to use the Braden PI Risk Assessment Tool	4.20	0.676	0.16
A123 Learn the technique of repositioning	4.13	0.743	0.18
A124 Learn the skill of position transfer.	4.20	0.676	0.16
A125 Learn to instruct the older adult on the use of walking aids.	4.73	0.594	0.13
A126 Learn the technique of wound dressing change.	4.07	0.884	0.22
A13 Attitude objectives	4.80	0.561	0.12
A131 Be able to respect and care for the older adult and love older adult care work	4.73	0.799	0.17
A132 Be able to understand the laws and regulations related to PI in nursing homes, and respect and protect the legal rights and interests of the older adult	4.87	0.352	0.07
A133 Through active participation in training courses, enhance self-efficacy, build the right attitude and confidence in preventing and managing PI	4.87	0.352	0.07
A134 Cultivate teamwork spirit, share experience and skills with colleagues, and jointly improve the quality of care	4.73	0.799	0.17
A135 Develop a high level of concern for the physical and mental health of the older adult, and always maintain patience, care and responsibility.	4.67	0.617	0.13
A136 Master the communication skills with nurses, the older adult, and their families, and provide psychological care for the older adult	4.80	0.414	0.09
B1 Training Content	4.80	0.414	0.09
B11 Theoretical knowledge module	4.67	0.617	0.13
B111 Professional ethics and code of conduct for nursing home caregivers	4.80	0.561	0.12
B112 Legal and ethical issues related to older adult care and PI	4.67	0.724	0.16
B113 Definition and epidemiological characteristics of PI	4.40	0.507	0.12
B114 The dangers of PI	4.80	0.561	0.12
B115 The mechanisms of occurrence and risk factors for PI	4.73	0.594	0.13
B116 Staging and clinical manifestations of PI	4.60	0.507	0.11
B117 Susceptible populations and common sites for PI	4.93	0.258	0.05
B118 Causes and characteristics of PI among the older adult in nursing homes	4.20	0.561	0.13
B119 Methods for risk assessment of PI	4.20	0.676	0.16
B1110 Methods for differentiating PI from other common skin conditions (such as incontinence-associated dermatitis, diabetic foot ulcers).	4.73	0.458	0.10
B1111 Nutrition care	4.60	0.737	0.16
B1112 Skin care	4.33	0.816	0.19
B1113 Treatment and management of PI	4.07	0.884	0.22
B1114 Types and functions of pressure relief equipment.	4.00	0.756	0.19
B1115 The selection and use of dressings	4.60	0.507	0.11
B1116 Psychological care for patients with PI	4.60	0.737	0.16
B1117 Communication skills for PI	4.53	0.743	0.16
B12 Practical Skills Module	4.67	0.617	0.13
B121 Repositioning skills	4.60	0.737	0.16
B122 Position transfer techniques	4.80	0.414	0.09
B123 Wound dressing change technique.	4.47	0.640	0.14
B124 Use of walking aids	4.20	0.414	0.10
B125 The selection and usage methods of dressings.	4.47	0.743	0.17
C1 Training methods	4.87	0.352	0.07
C11 Training format	4.73	0.594	0.13
C111 Online theoretical knowledge training (watching educational resources on the learning platform of the online course, setting a “no fast-forward” viewing learning function, and adding an “answering questions” function at important points of video knowledge, the platform can monitor the learning progress and learning effect of the students).	4.60	0.737	0.16
C112 Offline practical skills training	4.73	0.594	0.13
C12 Teaching methods	4.73	0.594	0.13
C121 Attention: Online self-learning by watching videos + offline learning by observing and imitating role models (expert workshops + case teaching);	4.73	0.458	0.10
C122 Retention: Online knowledge quizzes (insert “answering questions” function at important points in online teaching videos for quizzes to ensure learning effectiveness) + offline self-practice + offline situational simulation + offline peer learning + offline collaborative learning.	4.87	0.352	0.07
C123 Motor Reproduction: Knowledge feedback (theory and skills feedback and real-time feedback).	4.60	0.507	0.11
C124 Motivation: encouraging teaching (giving positive feedback), policy support (such as reduced workload, improved income, improved working conditions).	4.53	0.640	0.14
C13 Training time	4.53	0.640	0.14
C131 Online course training: Each video is <10 min long, with a total of 10–15 videos, and can be completed within 2 weeks	4.60	0.737	0.16
C132 Offline training: instructional demonstration for 2 h + self-directed practice in class for 1 h (to be completed within the same week) + self-directed practice after class for 2 h (to be completed within the second week), complete offline training and practice within 2 weeks.	4.60	0.632	0.14
D1 Training Evaluation	4.87	0.352	0.07
D11 knowledge evaluation	4.87	0.352	0.07
D111 Use the “PI Knowledge Questionnaire “designed by Zhou Dongmei, measuring the results pre-training and post-training. It consists of 25 items, 1 point for correctness, 0 point for otherwise, and the total scores range from 0 to 25 points. The higher the total score, the higher the level of PI knowledge.	4.87	0.352	0.07
D12 Practice evaluation	4.73	0.594	0.13
D121 Use the “PI Practice Questionnaire” designed by Zhou Dongmei, measuring the results pre-training and post-training. The scale consists of 20 items and 4 dimensions, including 10 items for repositioning behavior, 4 items for skin care, 4 items for wound care, and 2 items for nutritional support. Scoring criteria: “Never” is scored as “0,” “sometimes” is scored as “1,” “most of the time” is scored as “2,” “always” is scored as “3,” reverse items are scored in order from 3 to 0, The total score of the scale is 0–60 points. The higher the score, the better the caregiver’s implementation of caring practice.	4.73	0.458	0.10
D122 Use “PI identification skills questionnaire” (including PI staging and differentiation from other skin conditions) (self-designed); measuring the results pre-training and post-training. It is a set of photographs of different wound types. This will be measured by an assessment tool including a set of photographs depicting various PI stages (e.g., Stage I, II, III, IV, deep pressure tissue injury, unstageable, or medical device-related) of PIs and other skin conditions (such as diabetic foot ulcer, incontinence-associated dermatitis). The photographs are contributed by the researchers, and they are obtained from the clinical practice or Internet. The content validity of the instrument is validated by experts. The higher the total scores, the higher the degree of PI identification ability.	4.67	0.617	0.13
D13 Attitude evaluation	4.67	0.488	0.10
D131 Use the “PI Attitude Questionnaire”designed Zhou Dongmei, measuring the results pre-training and post-training. A total of eight items and items are scored from 1 to 4 points with the scores increasing from “strongly disagree,” “disagree,” “agree” and “strongly agree” for each item; The higher the score, the higher the level of PI prevention and management.	4.93	0.258	0.05
D132 Use the General Self-Efficacy Scale, measuring the results pre-training and post-training. There are 10 items in total, and each item is scored from 1 to 4. “completely incorrect” is scored as 1 point, “somewhat correct” is scored as 2 points, “mostly correct” is scored as 3 points, and “completely correct” is scored as 4 points. The higher the score, the stronger the self-confidence to deal with difficulties.	4.87	0.352	0.07
D14 Process evaluation	4.53	0.516	0.11
D141 During the training process, the online in-class tests and offline independent practice effects will be evaluated.	4.73	0.458	0.10
D15 satisfaction evaluation	4.87	0.352	0.07
D151 After the training, the nursing assistants evaluated their satisfaction and gave feedback on the training format, teaching methods, time schedule, teachers, and content, etc.	4.73	0.458	0.10

**Figure 2:**
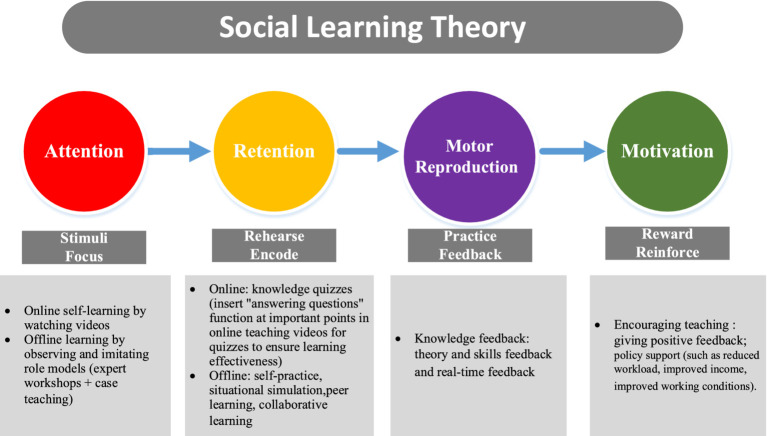
The process of SLT integrating into the PI training program intervention.

We demonstrated how the SLT integrated into the PI training program intervention in Figure 2.

## 4 Discussion

This study aimed to develop a comprehensive PI program for nursing assistants in Chinese nursing homes by leveraging SLT and refining the program through the modified Delphi method. The study highlights the significance of a methodical and theoretically based approach to training, which can significantly enhance the competencies of PIPM. The significance of PIPM in nursing homes is widely recognized by consumers and other stakeholders, assuming that the incidence and prevalence of PIs are indicators of poor nursing care quality (5). International multiple studies have shown that inappropriate care by caregivers contributes to the development and progression of PIs among patients. As the primary caregivers, nursing assistants normally face the following problems: low education level, lack of entry standard, non-professional, shortage, over workload, and low salaries, which can lead to problems including lack of basic nursing knowledge and skills, lack of regulation, unclear responsibilities, and heavy workloads, and impact nursing care service quality negatively (42–44). Therefore, the PI competency improvement training is very imperative and urgent. At present, the PI training for nursing assistants in nursing homes is not complete in China and even the global scope. The established SLTbT training program regarding PI provides a theoretical framework for global future study.

Expert opinions were gathered and a consensus was reached on the indicators of an effective SLTbT program through the modified Delphi method in this study. This method is particularly useful in situations where there is a need for structured communication among a group of experts to reach an agreement on complex issues (31, 45). The modified Delphi method was instrumental in achieving consensus among experts from various fields, including wound care, community nursing, geriatric nursing, and nursing education. The Kendall’s W of each round were 0.372 (*p* < 0.001, *χ*^2^ = 429.489), and 0.177 (*p* < 0.00 1, *χ*^2^ = 207.472) respectively. Based on the preset consensus criteria, all indicators reached a high degree of consensus (mean > 4.0; CV <0.25). The two-round Delphi process allowed for the systematic collection and refinement of expert opinions. The final PI training program for nursing assistants included 79 indicator items: 4 first-level indicators involving four domains (training objectives, training contents, training methods, training evaluation), 13 second-level indicators (three indicators for training objectives domain, two indicators for training contents domain, three indicators for training methods domain, five indicators for training evaluation domain), and 62 third-level indicators (25 indicators for training objectives domain, 22 indicators for training contents domain, eight indicators for training methods domain, seven indicators for training evaluation domain).

The developed PI training program for nursing assistants in Chinese nursing homes is underpinned by SLT, which posits that learning occurs through observation, imitation, and reinforcement of behaviors (29). SLT points out that combining the demonstration observation of real “role models” with individual autonomy and subjectivity can achieve individual self-learning and education (24). The integrated theory application was consistent with previous studies. Abdullah et al. (46) applied SLT to nursing trainees, effectively improving their professional abilities. This theoretical grounding is crucial as it provides a framework for understanding how nursing assistants can acquire the necessary knowledge and skills for PIPM through the training program. By incorporating SLT principles such as attention, retention, motor reproduction, and motivation, the training program ensures that nursing assistants not only acquire theoretical knowledge but also develop practical skills through observation and modeling. The proposed PIPM education model featured blended modules, interactive modules, simulation-based learning, peer-based learning, and case-based discussions. By observing and imitating the behavior of expert “role models” online and offline, the nursing assistants’ learning attention is aroused. In the form of peer learning and group cooperative learning, the nursing assistants can indirectly reflect on their learning behavior while observing the learning behavior of others, thus realizing alternative reinforcement of learning behavior (47). By using highly practical teaching methods such as case study discussions, scenario simulations, and repeated independent practice, learning behaviors are replicated, ensuring the effectiveness of the training. Combined with real-time feedback and absorption of knowledge and skills, this approach motivates learning and facilitates deep learning. Furthermore, this study innovates new strategies for nursing education by developing a training program to enhance nursing assistants’ competencies in PIPM based on SLT. This program is designed based on the actual training needs of nursing assistants in nursing homes and the need for policy support. It not only provides theoretical and skills-based support tailored to their training time or schedules, teaching methods, and evaluation methods but also addresses the challenges they face in terms of working conditions, workload, and income by offering policy support. This developed training program or education model may make a big difference in future nursing assistants’ training, education and cultivation in China, especially in the aspects of policy support such as improved working conditions, reduced workload, and improved income. These problems were also highlighted in previous studies in which nursing assistants complained they had poor working conditions, heavy workload, and low income (43, 44). In addition, this study may contribute to great implications for international clinical practice. The study advocates for policy incentives and emphasizes the synchronized development of nursing assistants’ knowledge, skills, and career development needs. In addition, it also strengthens nursing assistants’ vocational ethics and understanding of the vocational role, fostering the right professional values to cultivate high-competent nursing assistants. The content of the PI training program is different from previous studies globally. For example, Howe et al. (48) developed a PI educational program that focused on patient skin care through the following common training methods: PowerPoint slides, hands-on demonstrations, and group discussions (48). In demonstration skills, the nursing assistants practiced appropriate positioning, use of specialty beds, and off-loading pressure points (48). Cross et al. (49) designed a PI education program for residential care homes’ nursing assistants, and that just highlighted the basic knowledge and skills with the training method of a clinical nurse specialist’s 2 h lectures and did not give the competency evaluation finally. What is more, all previous studies constructed the program content based on outdated guidelines, and normally they did not measure the training effects toward competency improvement. Additionally, there is a scarcity of research that emphasizes training programs specifically addressing PI management or care, mostly focusing on PI prevention (50, 51). However, in the present study, the program content was designed based on the latest international guideline which involves basic concepts, risk factors, causes and mechanism, clinical stages, risk assessment methods, basic management and treatment methods, skin care, nutrition care, prevention skills, communication skills, psychological care, Legal and ethical issues, and so on.

In China, there is no national-level training program for nursing assistants in nursing homes (52). Nursing assistants’ careers start relatively late in China. While some LTC facilities implement occupational competency-strengthening programs, there is insufficient evidence to support the training effectiveness as it is not a standard, systemic approach and has little effect on PI knowledge and skills (43). Different nursing home has various training programs based on the Nursing Assistant Training Syllabus launched by the National Civil Bureau (53). The training syllabus includes daily care, basic care, rehabilitation services, psychological support, hospice care, care assessment, quality management and training guidance, and other related knowledge and skills (53). However, the training sessions on PI are very limited and sometimes account for only 1 h during the whole training program. Therefore, the current effects of PI training cannot be guaranteed. In addition, the limited PI training programs worldwide mainly target nurses, patients, family caregivers, or nursing assistants in hospitals, and the training curriculum quality, training content, training methods, and training evaluation varied greatly between these programs (43, 52, 54). Furthermore, these programs are often too broad and lack a specific focus on PIPM competencies, which are essential for providing high-quality care in nursing homes. There is a paucity of systemic and comprehensive PI-specific training programs focused on nursing assistants’ competencies in nursing homes worldwide. Therefore, this study has the potential to significantly enhance the care quality of residents in nursing homes by implementing the SLTbT program. It also has a broad and deep influence on international public health and clinical practice such as nursing assistant cultivation, policy-making, nursing management, public awareness improvement, aging population challenges, and so forth.

## 5 Limitations

It has several limitations in this study. Firstly, the study employed a modified Delphi method to achieve an agreement among an expert panel. Though it is effective for collective opinions, the limited number of experts may fail to represent the different ideas of all stakeholders, which might affect the generalizability of the findings. Secondly, the Delphi method based on experts’ opinions could cause subjective interpretation which may influence response accuracy. Thirdly, the study did not pilot test the training intervention with real-world situations that could have offered valuable feedback on program’s feasibility and efficacy.

## 6 Conclusion

This study successfully developed a theoretically grounded PI training program for nursing assistants in Chinese nursing homes. By integrating SLT and utilizing the modified Delphi method, the program addresses the critical need for effective PI training and represents a significant contribution to the field of geriatric care. The comprehensive training indicators provide a solid foundation for improving PIPM practices, ultimately enhancing the wellbeing of older adult residents. The program’s effectiveness has yet to be empirically tested in a real-world setting. Future research should focus on the program’s implementation and its impact on nursing assistants’ performance and older adult residents’ health outcomes.

## Data Availability

The original contributions presented in the study are included in the article/supplementary material, further inquiries can be directed to the corresponding authors.
